# Prevalence and subtype distribution of *Blastocystis* in ethnic minority groups on both sides of the China–Myanmar border, and assessment of risk factors

**DOI:** 10.1051/parasite/2019046

**Published:** 2019-07-25

**Authors:** Baiyan Gong, Xiaohua Liu, Yanchen Wu, Ning Xu, Meng Xu, Fengkun Yang, Lei Tong, Kexin Zhou, Jianping Cao, Aiqin Liu, Yujuan Shen

**Affiliations:** 1 Department of Parasitology, Harbin Medical University Harbin 150081 Heilongjiang PR China; 2 National Institute of Parasitic Diseases, Chinese Center for Disease Control and Prevention, Chinese Center for Tropical Diseases Research, WHO Collaborating Centre for Tropical Diseases, National Center for International Research on Tropical Diseases, Ministry of Science and Technology, Key Laboratory of Parasite and Vector Biology, MOH Shanghai 200025 PR China

**Keywords:** *Blastocystis*, humans, subtyping, risk factors, gastrointestinal symptoms

## Abstract

*Blastocystis* is one of the most common intestinal parasites in humans worldwide. To understand its prevalence and to explore the risk factors for *Blastocystis* in humans in developing countries, a molecular epidemiological investigation of *Blastocystis* was conducted in ethnic minority groups on both sides of the China–Myanmar border. A total of 461 fecal specimens were collected from 289 Yao people in China and 172 Wa people in Myanmar, together with a questionnaire for each participant. Based on sequence analysis of the partial small subunit of ribosomal RNA (SSU rRNA) gene (barcode region or 260 bp region), an average prevalence of 6.29% (29/461) was observed, with 4.50% (13/289) in Yao people and 9.30% (16/172) in Wa people. Twenty-two *Blastocystis* isolates were successfully subtyped by sequence analysis of the barcode region. Three subtypes were identified: ST1 (*n* = 7), ST3 (*n* = 13) and ST4 (*n* = 2). A statistical difference in the prevalence of *Blastocystis* was only observed between children (12.37%, 12/97) and adults (4.95%, 16/323), and between not washing hands (11.02%, 14/127) and washing hands (4.76%, 15/315) after using toilets, emphasizing the importance and necessity of health education in people in the investigated areas, especially in children.

## Abstract (fr)

*Blastocystis* est l’un des parasites intestinaux les plus répandus chez l’homme dans le monde. Pour comprendre la prévalence de *Blastocystis* et explorer les facteurs de risque chez l’homme dans les pays en développement, une enquête épidémiologique moléculaire sur *Blastocystis* a été menée auprès de groupes ethniques minoritaires des deux côtés de la frontière Chine-Myanmar. Au total, 461 spécimens fécaux ont été collectés auprès de 289 Yao en Chine et 172 Wa au Myanmar, accompagnés d’un questionnaire pour chaque participant. D’après l’analyse de la séquence du gène partiel de l’ARNr SSU (région du barcode ou région de 260 pb), une prévalence moyenne de 6,29 % (29/461) a été observée, dont 4,50 % (13/289) chez les Yao et 9,30 % (16/172) chez les Wa. Vingt-deux isolats de *Blastocystis* ont été sous-typés avec succès par analyse de séquence de la région du barcode. Trois sous-types ont été identifiés : ST1 (*n* = 7), ST3 (*n* = 13) et ST4 (*n* = 2). Une différence statistique dans la prévalence de *Blastocystis* n’a été observée qu’entre les enfants (12,37 %, 12/97) et les adultes (4,95 %, 16/323) et entre le fait de ne pas se laver les mains (11,02 %, 14/127) et se laver les mains (4,76 %, 15/315) après avoir utilisé les toilettes, ce qui souligne l’importance et la nécessité de l’éducation à la santé chez les habitants des zones étudiées, en particulier les enfants.

## Introduction

*Blastocystis* is a common eukaryotic microorganism worldwide, living in the large intestine of humans and numerous animal hosts including insects and reptiles [1]. Humans can be infected via the fecal-oral pathway, mainly through ingesting water and food contaminated with *Blastocystis* cysts as well as close-contact with animals infected with *Blastocystis* [11]. The number of individuals infected by this parasite is estimated to have increased to more than one billion worldwide [2]. The prevalences reported vary according to geographical regions but are generally higher in developing countries (22.1–100%) than in developed countries (0.5–23.1%), which might be related to differences in hygiene standards, waste disposal, exposure to animals, and consumption of contaminated food or water [29, 35].

Currently, multiple methods have been used for the detection of *Blastocystis*. Common direct smear examination by light microscopic and *in vitro* culture does not seem to reflect the true prevalence of *Blastocystis* in fecal specimens due to the low sensitivity and specificity of the diagnostic techniques employed [40, 44]. Molecular diagnostic techniques have been widely used for accurate identification and subtyping of *Blastocystis*, and the small subunit of ribosomal RNA (SSU rRNA) gene has been a popular gene marker for diagnostic Polymerase chain reaction (PCR) assays [40]. PCR followed by restriction fragment length polymorphism (PCR-RFLP) results are difficult to compare when different numbers and types of restriction endonucleases are used, or when the PCR products are amplified using different primer pairs [38]. PCR employing subtype-specific sequence-tagged-site primers (STS PCR) has the limitation of detecting only seven known subtypes, ST1–ST7 [6]. However, sequence analysis of PCR-amplified SSU rRNA gene fragments has the ability to both identify the presence of *Blastocystis* and genetically characterize this organism. To date, at least 17 subtypes (ST) have been identified in mammals and birds [11]. Ten subtypes (ST1–ST9 and ST12) have been reported in humans, with nine (ST1–ST8 and ST12) being potentially zoonotic [8]. Four subtypes (ST1–ST4) show the highest frequency (more than 90%) in human *Blastocystis* carriage, with a predominance of ST3 (around 60% of these isolates) [5]. In contrast, the other subtypes (ST5–ST8), rarely identified in humans, are more commonly found in some animal hosts: ST5 in hoofed animals, ST6 and ST7 in birds, and ST8 in non-human primates [39]. ST12 has been found in giraffes, kangaroos, yaks, waterbuck, cattle, and goats [32].

The pathogenic role of *Blastocystis* is controversial because of the presence of *Blastocystis* in significant numbers in both symptomatic and asymptomatic individuals, and the inconsistency of clinical symptoms [21]. Some studies suggested that *Blastocystis* carriage was associated with gastrointestinal symptoms (including diarrhea, abdominal pain, bloating and flatulence), irritable bowel syndrome (IBS) and extra-intestinal disorders (including urticaria, iron deficiency anemia and chronic angioedema) [1]. However, some studies have shown that *Blastocystis* can be a commensal parasite in humans without any pathogenic effect [14], and even appears to be more common in healthy individuals than in individuals with IBS [23]. Additionally, other studies considered *Blastocystis* a common member of the intestinal flora in healthy individuals [33].

Recently, some studies reported occurrence of *Blastocystis* in ethnic minority groups. Risk factors related to *Blastocystis* were explored in Orang Asli (Aborigine) in Malaysia [3, 24]. In a study on the molecular characterization of *Blastocystis* conducted in the Indigenous Tapirapé ethnic group from the Brazilian Amazon Region, the subtype distribution was observed to be markedly different from that reported in Europe: ST4 was not detected and ST3 was not the most common subtype [20]. However, little information is available on *Blastocystis* carriage in Yao people and Wa people from both sides of the China–Myanmar border. To understand the prevalence and subtype distribution of *Blastocystis* in these ethnic minority groups, a cross-sectional investigation of *Blastocystis* was conducted in the present study by PCR and sequence analysis of the partial SSU rRNA gene. Additionally, possible risk factors for *Blastocystis* carriage were assessed.

## Materials and methods

### Ethics statement

The present study was reviewed and approved by the Ethics Committee of the National Institute of Parasitic Diseases, Chinese Center for Disease Control and Prevention, China, and Myanmar Eastern Shan State Special Region 2 Ethic Health Organization. All participants were informed of the study objectives and the procedures involved in study participation at enrollment. Written informed consent was obtained from all adult participants before collection of fecal specimens. Additionally, for the individuals under 18 years of age, written consent was obtained from their guardians.

### Specimen collection

During the period between April and October 2018, a cross-sectional investigation of *Blastocystis* was carried out in seven villages in two study sites on both sides of the China–Myanmar border. Approximately 5–10 g of fresh fecal specimens were collected from 461 people (one each). In all, 62.69% (289/461) of specimens were from Yao people (aged 21–72 years) in three villages of the Yao Ethnic Township in Mengla County of Xishuangbanna Dai Autonomous Prefecture, Yunnan Province, China (101.33 °E and 21.27 °N). Similarly, 37.31% (172/461) of specimens were from Wa people (aged 7–53 years) in four villages in Pangkham, a main town of Pangsang Township of Matman District, Shan State, Myanmar (99.11 °E and 22.10 °N). At the time of sampling, diarrheal specimens were noted to have loose or liquid stools by macroscopic observation, whereas the remaining had normal consistency and color. All the specimens were delivered to the laboratory in a cooler with ice packs within 24 h after collection and stored in a freezer at –20 °C before DNA extraction.

### Questionnaire

A structured questionnaire was administered to each study participant by health professionals from the local Center for Disease Control and Prevention (CDC). The questionnaire contained some information on socio-demographic characteristics, personal hygiene habits, and other possible risk factors for *Blastocystis* carriage as well as common gastrointestinal symptoms (diarrhea, nausea, emesis, abdominal pain and anorexia) (S1 Questionnaire). Meanwhile, each questionnaire was linked to one fecal specimen and used for our analysis in the present study.

### DNA extraction

Genomic DNA was directly extracted from approximately 180–200 mg of each of fecal specimen using a QIAamp DNA Stool Mini Kit (QIAgen, Hilden, Germany), according to manufacturer-recommended procedures and the provided reagents. Meanwhile, to obtain a high yield of DNA, the lysis temperature was increased to 95 °C. DNA was eluted in 200 μL of AE and stored at −20 °C prior to use in molecular analysis.

### PCR amplification

All DNA preparations were identified and subtyped for *Blastocystis* by amplifying a 600 bp nucleotide fragment (barcode region) of the SSU rRNA gene of *Blastocystis* [34]. To increase the detection rate of *Blastocystis*, all negative DNA preparations in the barcode region were subsequently subjected to PCR amplification of a nucleotide fragment of approximately 260 bp within the barcode region, an amplicon that only allows detection of the parasite [22]. The primers and the cycling parameters were used as described previously by Menounos et al. [22] and Scicluna et al. [34].

TaKaRa Taq DNA polymerase (TaKaRa Bio Inc., Tokyo, Japan) was used for all PCR reactions. A negative control without DNA and a positive control (DNA of *Blastocystis* ST10 derived from a sika deer) were used in all PCR tests. Each DNA preparation was analyzed at least twice by PCR. All PCR products were subjected to electrophoresis in a 1.5% agarose gel and visualized by staining the gel with GelStrain (TransGen Biotech., Beijing, China).

### Nucleotide sequencing and analyzing

All the positive PCR products of expected size were directly sequenced with their respective PCR primers on an ABI PRISM™ 3730 DNA Analyzer by Sinogeno-max Biotechnology Co., Ltd. (Beijing, China), using a BigDye Terminator v3.1 Cycle Sequencing kit (Applied Biosystems, Foster City, CA, USA). Sequence accuracy was confirmed by two-directional sequencing and by sequencing two additional PCR products for some DNA preparations, which produced the sequences different from those published in GenBank databases. Nucleotide sequences obtained in the present study were compared to all *Blastocystis* homologous sequences published in GenBank using BLAST searches (http://www.ncbi.nlm.nih.gov/blast/). They were then aligned and analyzed with each other and reference sequences downloaded from the GenBank database using the program Clustal X 1.83 (http://www.clustal.org/) to determine the presence and subtypes of *Blastocystis*. *Blastocystis* subtypes were identified by determining the exact match or the closest similarity according to the proposed standard of *Blastocystis* terminology [38].

Two novel nucleotide sequences obtained in the present study were deposited in the GenBank database under the following accession numbers: MK898939 and MK898940.

### Statistical analyses

Statistical analyses (Pearson chi-square test or Fisher’s exact test) were performed using the Statistical Package for the Social Sciences (SPSS) 19.0 to assess possible risk factors for *Blastocystis* carriage (Table 1), and to determine the relationship between *Blastocystis* carriage and gastrointestinal symptoms (Table 2), respectively. A *p* value of < 0.05 was considered statistically significant.

**Table 1:** Assessment of possible risk factors for *Blastocystis* carriage.

Variable	No. positive/No. examined^b^ (%)	OR (95% CI)^c^	χ^2^/*p* value
Gender			
Male	12/247 (4.86)	0.59 (0.28, 1.27)	1.85/0.17
Female	17/214 (7.94)		
Age (years)			
Children (7–12)	12/97 (12.37)	Ref	
Teenagers (13–17)	1/41 (2.44)	5.65 (0.71, 44.95)	2.27/0.13
Adults (≥18)	16/323 (4.95)	0.37 (0.17, 0.81)	**6.60/0.01** d
Drinking boiled water			
Yes	25/393 (6.36)	1.31 (0.44, 3.93)	0.03/0.86
No	4/49 (8.16)		
Washing hands before meals			
Yes	17/322 (5.28)	1.99 (0.92, 4.31)	3.18/0.08
No	12/120 (10.00)		
Washing hands after using toilets			
Yes	15/315 (4.76)	2.48 (1.16, 5.30)	**5.79/0.02** d
No	14/127 (11.02)		
Eating unwashed vegetables and fruits			
Yes	25/381 (6.56)	1.00 (0.34, 2.98)	0/1.00
No	4/61 (6.56)		
Swimming^a^			
Yes	2/40 (5.00)	1.37 (0.31, 5.98)	0.01/0.93
No	27/402 (6.72)		
Pit toilets			
Public	25/364 (6.87)	1.36 (0.46, 4.04)	0.10/0.76
Individual	4/78 (5.13)		
Animal feeding patterns			
Free-ranging	6/86 (6.98)	1.52 (0.41, 5.74)	0.07/0.79
Both free-ranging and captive	19/317 (5.99)	0.56 (0.18, 1.73)	0.46/0.50
Captive	4/39 (10.26)	Ref	

a Ponds are the only swimming places in the investigated areas.

b Only 442 participants provided complete information (other than gender and age).

c CI, confidence interval.

d Bold type for values indicates statistical significance.

**Table 2:** Relationship between *Blastocystis* carriage and gastrointestinal symptoms.

Group	No. positive/No. examined (%)					
	Gastrointestinal symptoms^a^	Diarrhea	Abdominal pain	Nausea	Emesis	Anorexia
*Blastocystis*-positive	6/29 (20.69)	6/29 (20.69)	2/29 (6.90)	0/29	0/29	0/29
*Blastocystis*-negative	92/432 (21.30)	79/432 (18.29)	38/432 (8.80)	7/432 (1.62)	12/432 (2.78)	10/432 (2.31)
OR (95% CI)	0.96 (0.38, 2.44)	1.17 (0.46, 2.96)	0.77 (0.18, 3.36)	1.02 (1.00, 1.03)	1.03 (1.01, 1.05)	1.02 (1.00, 1.04)
χ^2^/*p* value	0.01/0.94	0.10/0.75	0/0.99	*–*/1.00^b^	*–*/1.00^b^	*–*/1.00^b^

a Indicating people who had at least one of the five symptoms listed in the present study.

b Fisher’s exact test.

## Results

### Prevalence of *Blastocystis* and possible risk factors

**Figure 1:**
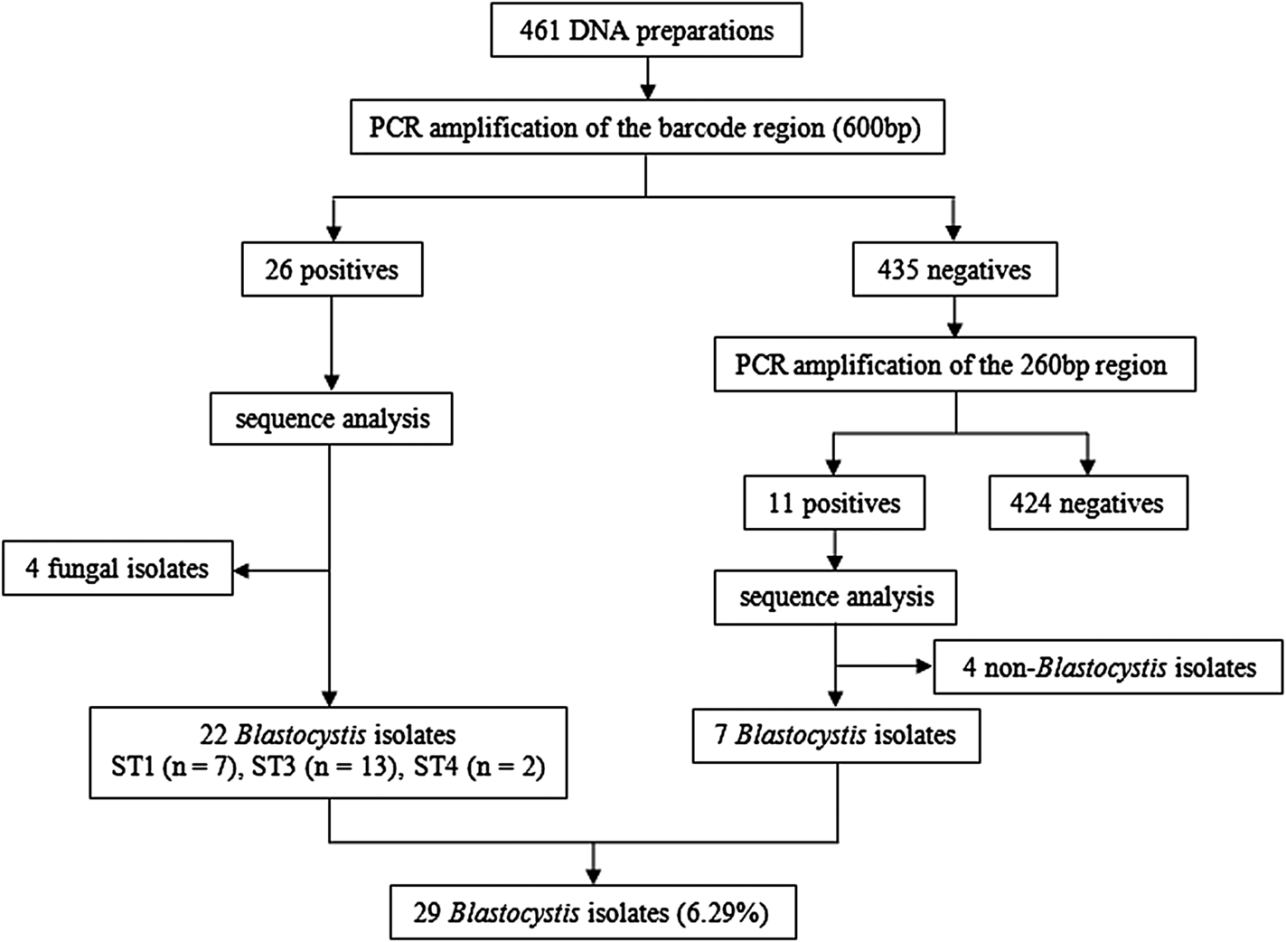
Flow diagram of prevalence and subtype identification of *Blastocystis*.

A total of 37 DNA preparations were successfully amplified and sequenced in either of the barcode regions and the 260 bp region of the SSU rRNA gene. Based on sequence analysis, 29 DNA preparations were confirmed to be positive for *Blastocystis* (Fig. 1). An average prevalence of 6.29% (29/461) was observed in the investigated people on both sides of the China–Myanmar border, with 4.50% (13/289) in Yao people in China and 9.30% (16/172) in Wa people in Myanmar. *Blastocystis* was found in all seven villages, with the prevalence ranging from 3.23% to 15.63% (Table 3).

**Table 3:** Prevalence and subtype distribution of *Blastocystis* in humans.

Location	No. positive/No. examined (%)	Subtype (*n*)^a^	GenBank ID^b^	
			Human	Animal (species)
Yunnan, China				
Village 1	3/93 (3.23)	ST3 (2)	KY610153	AB107963 (Pig); AB107965 (Cattle); KY929101 (Macaque)
		Unknown (1)		
Village 2	4/110 (3.64)	ST1 (1)	MH349749	MF186699 (Goat); AB107967 (Orang-utan)
		ST3 (1)	MF184939	
		ST3 (2)	KY610153	AB107963 (Pig); AB107965 (Cattle); KY929101 (Macaque)
Village 3	6/86 (6.98)	ST1 (1)	MF184949	MH021854 (Brown rat)
		ST1 (1)	KY610125	KY610167 (Pig)
		ST3 (2)	MF184939	
		**ST3 (1)**	MK898939	
		**ST4 (1)**	MK898940	
Subtotal	13/289 (4.50)	ST1 (3), ST3 (8), ST4 (1), Unknown (1)		
Shan, Myanmar				
Village 4	5/32 (15.63)	ST1 (3)	MH349749	MF186699 (Goat); AB107967 (Orang-utan)
		ST3 (1)	MF184939	
		Unknown (1)		
Village 5	4/52 (7.69)	ST3 (1)	KY610153	AB107963 (Pig); AB107965 (Cattle); KY929101 (Macaque)
		ST4 (1)	MH197686	
		Unknown (2)		
Village 6	4/52 (7.69)	ST1 (1)	MH349749	MF186699 (Goat); AB107967 (Orang-utan)
		ST3 (1)	KY610153	AB107963 (Pig); AB107965 (Cattle); KY929101 (Macaque)
		Unknown (2)		
Village 7	3/36 (8.33)	ST3 (2)	KY610153	AB107963 (Pig); AB107965 (Cattle); KY929101 (Macaque)
		Unknown ST (1)		
Subtotal	16/172 (9.30)	ST1 (4), ST3 (5), ST4 (1), Unknown (6)		
Total	29/461 (6.29)	ST1 (7), ST3 (13), ST4 (2), Unknown (7)		

a Unknown indicates preparations in which DNA was only amplified and sequenced successfully in approximately 260 bp region of the SSU rRNA gene of *Blastocystis*.

b Accession No. indicating the sequences downloaded from GenBank, which have 100% homology with the sequences obtained in the present study except two novel sequences (highlighted in bold) obtained here.

In the present study, all 461 participants were asked to fill out questionnaires. However, 19 participants provided incomplete information except gender, age and gastrointestinal symptoms. In the end, 442 completed questionnaires were used for assessment of possible risk factors for *Blastocystis* carriage. The prevalences of *Blastocystis* were statistically different in two categories of possible risk factors: children (12.37%, 12/97) versus adults (4.95%, 16/323), and not washing hands after using toilets (11.02%, 14/127) versus washing hands after using toilets (4.76%, 15/315) (Table 1).

### *Blastocystis* subtypes and homology analysis

Twenty-two of 29 DNA preparations positive for *Blastocystis* were successfully subtyped by sequence analysis of the barcode region. Three subtypes were identified: ST1 (*n* = 7), ST3 (*n* = 13) and ST4 (*n* = 2) (Fig. 1). ST3 was the most common in people in the investigated areas (59.09%, 13/22). Three subtypes could be seen in both Yao people and Wa people (Table 3).

By homology analysis of the barcode region of 22 *Blastocystis* isolates, eight representative sequences were obtained, with three, three and two from ST1, ST3 and ST4 isolates, respectively. Among them, the two sequences identified as ST3 (MK898939) and ST4 (MK898940) were not described previously, but had the largest similarity with those in humans from Iran (MG011638) and Germany (AY244620), respectively.

Among the remaining six sequences, two have only been reported in humans previously, and four have also been identified previously in animals, along with humans. One ST1 sequence showed 100% identity with those in animals, including a goat from the UK (MF186699) and an orang-utan from Japan (AB107967). Another two ST1 sequences were identical to those in a brown rat from the Czech Republic (MH021854) and a pig from the Philippines (KY610167), respectively. One ST3 sequence shared 100% sequence identity with those in animals, including a pig from the Philippines (KY610169) and Japan (AB107963), a cow from Japan (AB107965) and a macaque from the Philippines (KY929101) (Table 3).

### Relationship between *Blastocystis* carriage and gastrointestinal symptoms

All 461 questionnaires were used for assessment of the relationship between *Blastocystis* carriage and gastrointestinal symptoms. No statistical relationship was observed between them, accounting for 6.34% (23/363) and 6.12% (6/98) in asymptomatic and symptomatic individuals, respectively (Table 2).

## Discussion

*Blastocystis* has been reported in humans worldwide. In the present study, 4.50% (13/289) of Yao people in China and 9.30% (16/172) of Wa people in Myanmar were confirmed to be infected with *Blastocystis*.

The prevalence of *Blastocystis* is reported to be related to multiple factors. The immune status of hosts seems to be one main risk factor for *Blastocystis* carriage [47]. Immunodeficient/immunocompromized populations are more susceptible to *Blastocystis* carriage and its associated symptoms, showing opportunistic pathogenesis [40]. Kurniawan et al reported a high prevalence (72.4%) of *Blastocystis* in HIV/AIDS patients presenting with diarrhea in Jakarta, Indonesia [15]. The present low prevalence of *Blastocystis* might be related to the large percentage (70.07%, 323/461) of specimens from immunocompetent adults. Diagnostic methods also affect the prevalence estimates of *Blastocystis*. PCR-based molecular methods are observed to be superior to non-molecular methods in sensitivity and specificity [38]. In North Cyprus, the prevalence of *Blastocysti*s was found to be 10.5%, 10.5%, and 27.8% in humans by direct microscopy, trichrome method, and PCR, respectively [35]. In another study conducted in school-aged children (age 12–54 months) in Colombia, the prevalence of *Blastocystis* was 25.19% by microscopy and 39.22% by qPCR [42]. In addition, poor hygiene habits possibly increase the opportunity of *Blastocystis* carriage. A cross-sectional survey of *Blastocystis* in Malaysia discovered by multivariate analysis that drinking untreated water and the presence of other family members infected with *Blastocystis* were significant risk factors among three Orang Asli tribes and the overall population studied [3]. However, in the present study, a statistical difference in the prevalence of *Blastocystis* was observed between children (12.37%, 12/97) and adults (4.95%, 16/323), and between not washing hands after using toilets (11.02%, 14/127) and washing hands after using toilets (4.76%, 15/315). Like other intestinal parasites, not washing before eating and after using the toilet as well as sucking their fingers for some children favors *Blastocystis* by fecal-oral transmission.

Current molecular epidemiological data have revealed that ST1–ST4 account for more than 90% of human cases of *Blastocystis* infection [8]. ST1 and ST3 are highly prevalent in Australia, Europe, and South Eastern Asia, while ST1 and ST2 are prevalent in America, and ST4 in Europe [36]. In recent studies from South America, ST1–ST3 were detected as the most frequent [30, 31]. However, ST3 was not the most common subtype in the Indigenous Tapirapé ethnic group in Brazil, which was considered to be related to little contact between indigenous groups and people in other communities [20]. In the present study, three subtypes (ST1, ST3 and ST4) were identified in two ethnic groups, and ST3 was the most common in Yao people (8/12, 66.67%) and in Wa people (5/10, 50%), which was similar to findings previously reported in almost all the studies in China (Table 4) [18, 19, 41, 49, 50, 53–57], and in some studies in a nearby country (Thailand) [25, 28, 37, 52]. Minor subtypes (ST5–ST9 and ST12) that are only very rarely found in humans also show differences in geographical distribution. ST6 and ST7 are common in Asia but rarely observed in European countries, and ST5, ST8, ST9 and ST12 are found occasionally in humans [30, 47]. This being the first subtyping report of *Blastocystis* in Myanmar, the constitution and ratio of *Blastocystis* subtypes in this country needs to be assessed in the future by systematic molecular epidemiological investigations in larger populations from more areas.

**Table 4:** Prevalence and subtype distribution of *Blastocystis* in humans by province in China.

Province	No. positive/No. examined (%)	Subtype (%)^c^													Refs.
		ST1	ST2	ST3	ST4	ST5	ST6	ST7	ST12	ST1 + 2	ST1 + 3	ST2 + 3	ST3 + 5	Unknown^c^	
Shanghai	29/1505 (1.93)	6 (20.7)	1 (3.4)	17 (58.7)	–	–	1 (3.4)	–	–	–	2 (6.9)	–	–	2 (6.9)	[18]
Zhejiang	10/170 (5.88)	3 (30.0)	1 (10.0)	6 (60.0)	–	–	–	–	–	–	–	–	–	–	[18]
Jiangxi	35^a^	13 (37.1)	2 (5.7)	14 (40.0)	–	–	–	–	–	–	5 (14.3)	–	–	1 (2.9)	[49]
	3^a^	–	–	–	–	2 (66.7)	–	–	–	–	–	–	1 (33.3)	–	[50]
Guangxi	1a,b	–	–	1 (100)	–	–	–	–	–	–	–	–	–	–	[53]
	10^a^	8 (80.0)	–	–	–	–	–	–	–	–	–	–	–	2 (20.0)	[54]
	53^a^	4 (7.6)	–	17 (32.1)	4 (7.5)	–	1 (1.9)	5 (9.4)	–	–	–	–	–	22 (41.5)	[55]
Yunnan	58/1440 (4.03)^b^	57 (98.3)	1 (1.7)	–	–	–	–	–	–	–	–	–	–	–	[56]
	12/324 (3.70)^b^	3 (25.0)	–	2 (16.7)	3 (25.0)	–	–	3 (25.0)	1 (8.3)	–	–	–	–	–	[41]
	153/646 (23.68)	38 (24.8)	7 (4.5)	93 (60.8)	1 (0.7)	–	–	–	–	1 (0.7)	6 (3.9)	1 (0.7)	–	6 (3.9)	[18]
	78/239 (32.64)	16 (20.5)	1 (1.3)	55 (70.5)	1 (1.3)	–	–	–	–	1 (1.3)	1 (1.3)	–	–	3 (3.8)	[19]
	13/289 (4.50)	3 (23.1)	–	8 (61.5)	1 (7.7)	–	–	–	–	–	–	–	–	1 (7.7)^d^	This study
Heilongjiang	27/381 (7.09)^b^	12 (44.4)	–	15 (55.6)	–	–	–	–	–	–	–	–	–	–	[57]
Total		160 (34.1)	13 (2.8)	220 (47.0)	9 (1.9)	2 (0.4)	2 (0.4)	8 (1.7)	1 (0.2)	2 (0.4)	14 (3.0)	1 (0.2)	1 (0.2)	36 (7.7)	

–: negative results.

a Only *Blastocystis* isolates were subtyped in some studies.

b These *Blastocystis* isolates were identified and subtyped by PCR and sequencing, while others were subtyped by STS PCR.

c Unknown subtypes of *Blastocystis* appearing in some studies were subtyped by STS PCR.

d This DNA preparation was only amplified and sequenced successfully in approximately 260 bp of the SSU rRNA gene of *Blastocystis*.

In fact, difference in constitutions and ratios of *Blastocystis* subtypes might be related to different epidemiological characteristics, i.e., reservoirs and routes of transmission. *Blastocystis* has been found in various animals. The risk of zoonotic transmission of *Blastocystis* has also been predicted by some previous studies. The same subtypes have been found in animals and their close contacts: ST1 and ST2 in zoo keepers and one wombat and five primate species in Australia [26]; ST2 in children and monkeys in Nepal [51]; ST5 in piggery workers and pigs in Australia [46]; ST6 in breeders and cattle/goats in Nepal [16, 17] and in slaughterhouse staff members and chickens in Lebanon [9]. Three subtypes (ST1, ST3 and ST4) of *Blastocystis* obtained in the present study have also been found in various animals, with ST1 in non-human primates (apes and baboons), monkeys, cattle, pigs, sheep, goats, foxes, dogs, and birds; ST3 in non-human primates, pigs, cattle, sheep, goats and racoon dogs; ST4 in rodents, rabbits, foxes, giraffes, kangaroos, dogs, a snow leopard, ostriches as well as non-human primates (ring-tailed lemurs, woolly monkeys and siamangs) [36, 44, 45]. More importantly, some ST1 and ST3 sequences obtained in the present study were identical to those from animals (Table 3), implying a possibility of zoonotic transmission of *Blastocystis*. However, due to the lack of data of *Blastocystis* in local animals, the epidemiologic role of animals in the spread of *Blastocystis* remains unclear. It is necessary in the future to collect animal fecal specimens to test this assumption, especially common domestic pigs, dogs and chickens kept near houses. If these animals are infected with *Blastocystis*, they increase the opportunity of human carriage; meanwhile, animal feces can enter streams and rivers through surface run-off after heavy rain, which causes water contamination downstream and wide geographical spread. To the best of our knowledge, two waterborne outbreaks of blastocystosis have been documented worldwide [10, 48]. A total of 1122 people were involved in one outbreak in China, highlighting the importance of *Blastocystis* in public health [48].

*Blastocystis* has been reported to give rise to gastrointestinal symptoms, like diarrhea, abdominal pain, flatulence, nausea, vomiting, constipation, weight loss or fatigue, and in addition, there is an association between *Blastocystis* and IBS as well as urticaria [36]. However, *Blastocystis* carriage is also frequently detected in asymptomatic individuals, implying a nonpathogenic effect [14]. In the present study, no associations were observed between *Blastocystis* carriage and gastrointestinal symptoms as well as each symptom category. Currently, no consensus has been reached over the pathogenicity of *Blastocystis*. Even though *Blastocysti*s shows pathogenicity in some studies, besides immune status of individuals, the occurrence and the severity of clinical symptoms are also observed to be related to the density of this pathogen in the intestinal tract as well as the virulence of subtypes [21, 40]. In a study conducted in Egypt, higher densities of *Blastocystis* were found in symptomatic patients than in asymptomatic ones with a statistically significant difference (8.2 cells vs. 3.8 cells/100 × field), and the mean number of *Blastocystis* was significantly higher in patients with diarrhea and abdominal pain [7]. Some studies have suggested a relationship between clinical symptoms and *Blastocystis* subtypes: STs 1, 2, 4 and 6 for gastrointestinal symptoms [4, 13]; ST2 for gastrointestinal symptoms and chronic urticaria [43]; and STs 1, 3 and 7 for IBS [12, 27]. In the present study, since 24.14% (7/29) of *Blastocystis* isolates were not successfully subtyped, the relationship between gastrointestinal symptoms and *Blastocystis* subtypes was not assessed.

## Conclusions

The present study described the occurrence of *Blastocystis* in ethnic minority groups on both sides of the China–Myanmar border. Like in other intestinal protozoan infections including *Giardia intestinalis* and *Entamoeba histolytica*, young age and not washing hands after using toilets were two risk factors for *Blastocystis* carriage in the investigated areas, emphasizing the importance and necessity of health education among local people, especially children. Although no association was observed between *Blastocystis* carriage and gastrointestinal symptoms here, the pathogenic potential of *Blastocystis* cannot be excluded.
