# Supplementary material for: Prevalence and subtype distribution of Blastocystis in ethnic minority groups on both sides of the China–Myanmar border, and assessment of risk factors
Source: Parasite. 2019 Jul 25;26:46. doi: 10.1051/parasite/2019046 (PMC6658150; doi:10.1051/parasite/2019046)
Supplement: Supplementary file 1 [file parasite-26-46-olm.pdf]

Questionnaire

First. Personal Information

1. Name:
2. Gender:
3. Age:
4. Tel:
5. Home address:

Second. Personal Habits

1. Water Habits

1.1 The source of drinking water

(1) Tap water (2) Well water (3) Barreled water (4) River/Lake water (5) Others

1.2 Drinking boiled water

(1) Yes (2) No

2. Eating Habits

2.1 Eating unwashed vegetables and fruits

(1) Yes (2) No

3. Hygienic habits

3.1 Washing hands before meals

(1) Yes (2) No

3.2 Washing hands after using toilets

(1) Yes (2) No

Third. Animals

1. Animal species raised

(1) Chickens (2) Pigs (3) Cattle (4) Sheep/Goats (5) Dogs (6) Rabbits (7) Others (species)

2. Feeding patterns

(1) Free-ranging (2) Captive (3) Both free-ranging and captive

3. Frequent contact with animals

(1) Yes (2) No

Fourth. Others

1. Pit toilets

(1) Public (2) Individual

2. Swimming (Only ponds can be used for swimming in the investigated areas)

(1) Yes (2) No

Fifth. Gastrointestinal Symptoms

1. Diarrhea

(1) Yes (2) No

2. Abdominal pain

(1) Yes (2) No

3. Nausea

(1) Yes (2) No

4. Emesis

(1) Yes (2) No

5. Anorexia

(1) Yes (2) No
